# Supplementary material for: Trans-regulation of heterochromatin underlies genetic variation in 3D genome contacts
Source: bioRxiv. 2025 Dec 16:2025.12.10.693515. Preprint. [Version 2] doi: 10.64898/2025.12.10.693515 (PMC12712905; doi:10.64898/2025.12.10.693515)
Supplement: Supplement 2 [file media-2.pdf]

MiniMUGA Background Analysis v2.3.1

Sample ID

13-841

Neogen ID

AAAS-5958

Summary

The genotype of this sample is of **excellent** quality. It is **male** and **inbred**, and likely a mix of **C57BL/6J** and **DBA/2J**.

Diagnostic SNPs are likely explained by the presence of the background strains

- Solution 1: C57BL/6J and DBA/2J
  - C57BL/6J: 165 / 170 (97.1%)
  - DBA/2J: 3 / 140 (2.1%)

No genetic constructs were detected in this sample.

Genotyping Quality

Excellent (2 N calls)
All reported results are dependent on genotyping quality.

Chromosomal Sex

XY

Inbreeding Estimate

100.0% Inbred
(Percentage of the genome (autosomal and X chromosomes) that is homozygous or hemizygous for primary, secondary, and unknown backgrounds. See Genome Analysis)

Constructs Detected

BlastR	bpA	Cas9	chlor	cHS4	Cre	DTA	Flp	g_FP	hCMV_a	hCMV_b	hTK_pr	iCre	IRES	Luc	r_FP	rtTA	SV40	tTA
-	-	-	-	-	-	-	-	-	-	-	-	-	-	-	-	-	-	-

Refined Ideogram

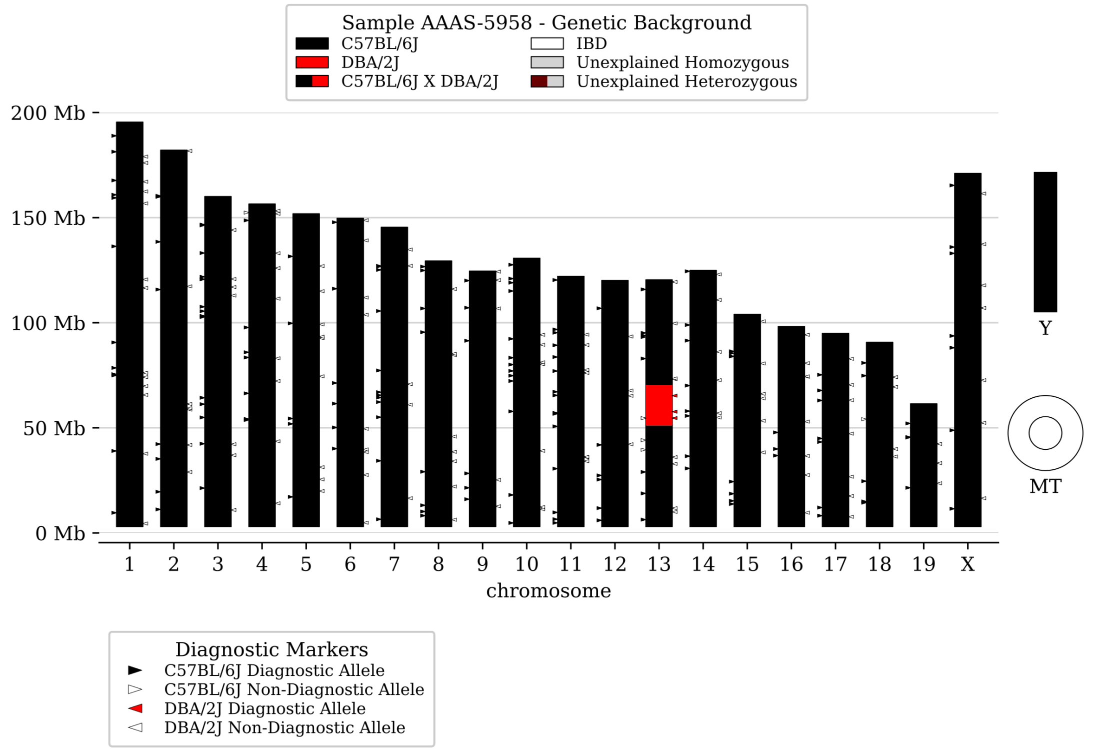

Genome Analysis

Background	Zygosity	Informative Markers	Informative Markers %	Genome %
C57BL/6J	Homozygous	2887	99.4%	99.2%
DBA/2J	Homozygous	16	0.6%	0.8%
	**Total**	2903	100.0%	100.0%

Y Chromosome

Y Haplogroup 11 - 100.0% Consistent
Includes C57BL/6J and 2 other strains

MiniMUGA Background Analysis v2.3.1

**MT Genome**	MT Haplogroup 6 - 100.0% Consistent<br>Includes C57BL/6J, DBA/2J and 166 other strains

Backgrounds Detected (Diagnostic Alleles)

	Diagnostic Alleles Observed			
**Diagnostic Class**	**Homozygous**	**Heterozygous**	**Potential**	**% Observed**
C57BL/6J, C57BL/6JJicTac, C57BL/6JRj	101	0	102	99.0%
C57BL/6J, C57BL/6JRj	29	1	31	96.8%
C57BL/6J, C57BL/6JEiJ, C57BL/6JJicTac, C57BL/6JRj	20	0	21	95.2%
B6N-Tyr<c-Brd>/BrdCrCrl, C57BL/6J, C57BL/6JJicTac, C57BL/6JRj	5	0	5	100.0%
C57BL/6J	3	0	5	60.0%
B6N-Tyr<c-Brd>/BrdCrCrl, C57BL/6J, C57BL/6JBomTac, C57BL/6JEiJ, C57BL/6JJicTac, C57BL/6JOlaHsd, C57BL/6JRj	2	0	2	100.0%
C57BL/6J, C57BL/6JBomTac, C57BL/6JEiJ, C57BL/6JJicTac, C57BL/6JOlaHsd, C57BL/6JRj	2	0	2	100.0%
DBA/2J	2	0	117	1.7%
B6N-Tyr<c-Brd>/BrdCrCrl, C57BL/6J, C57BL/6JEiJ, C57BL/6JJicTac, C57BL/6JRj	1	0	1	100.0%
C57BL/6J, C57BL/6JEiJ, C57BL/6JJicTac, C57BL/6JOlaHsd, C57BL/6JRj	1	0	1	100.0%
DBA/2J, DBA/2JRj	1	0	23	4.3%

Minimal Strain Sets Explaining All Diagnostic Classes (Number of Markers Explained):

- Solution 1: C57BL/6J and DBA/2J
    - C57BL/6J: 165 / 170 (97.1%)
    - DBA/2J: 3 / 140 (2.1%)

MiniMUGA Background Analysis v2.3.1

	Chromosome	Start (Mb)	Stop (Mb)	Background	Zygosity
**Diplotype Intervals**	1	3000000	195471971	C57BL/6J	Homozygous
	2	3000000	182113224	C57BL/6J	Homozygous
	3	3000000	160039680	C57BL/6J	Homozygous
	4	3000000	156508116	C57BL/6J	Homozygous
	5	3000000	151834684	C57BL/6J	Homozygous
	6	3000000	149736546	C57BL/6J	Homozygous
	7	3000000	145441459	C57BL/6J	Homozygous
	8	3000000	129401213	C57BL/6J	Homozygous
	9	3000000	124595110	C57BL/6J	Homozygous
	10	3000000	130694993	C57BL/6J	Homozygous
	11	3000000	122082543	C57BL/6J	Homozygous
	12	3000000	120129022	C57BL/6J	Homozygous
	13	3000000	51136860	C57BL/6J	Homozygous
	13	51136860	70449416	DBA/2J	Homozygous
	13	70449416	120421639	C57BL/6J	Homozygous
	14	3000000	124902244	C57BL/6J	Homozygous
	15	3000000	104043685	C57BL/6J	Homozygous
	16	3000000	98207768	C57BL/6J	Homozygous
	17	3000000	94987271	C57BL/6J	Homozygous
	18	3000000	90702639	C57BL/6J	Homozygous
	19	3000000	61431566	C57BL/6J	Homozygous
	X	3000000	171031299	C57BL/6J	Hemizygous
	Y	0	0	C57BL/6J	Hemizygous
	MT	0	0	IBD	Hemizygous
