# Supplementary material for: Trans-regulation of heterochromatin underlies genetic variation in 3D genome contacts
Source: bioRxiv. 2025 Dec 16:2025.12.10.693515. Preprint. [Version 2] doi: 10.64898/2025.12.10.693515 (PMC12712905; doi:10.64898/2025.12.10.693515)
Supplement: Supplement 3 [file media-3.pdf]

MiniMUGA Background Analysis v2.3.1

**Sample ID**	14-846
**Neogen ID**	AAAS-5953

Summary

The genotype of this sample is of **excellent** quality. It is **male** and **inbred**, and likely a mix of **DBA/2J** and (**C57BL/6J and/or C57BL/6JJicTac and/or C57BL/6JEiJ and/or C57BL/6JRj**).

Diagnostic SNPs are likely explained by the presence of the background strains

- Solution 1: C57BL/6J and DBA/2J
  - DBA/2J: 140 / 143 (97.9%)
  - C57BL/6J: 1 / 21 (4.8%)
- Solution 2: C57BL/6JJicTac and DBA/2J
  - DBA/2J: 140 / 143 (97.9%)
  - C57BL/6JJicTac: 1 / 21 (4.8%)
- Solution 3: C57BL/6JEiJ and DBA/2J
  - DBA/2J: 140 / 143 (97.9%)
  - C57BL/6JEiJ: 1 / 21 (4.8%)
- Solution 4: C57BL/6JRj and DBA/2J
  - DBA/2J: 140 / 143 (97.9%)
  - C57BL/6JRj: 1 / 21 (4.8%)

No genetic constructs were detected in this sample.

**Genotyping Quality**	**Excellent (6 N calls)**<br>All reported results are dependent on genotyping quality.
**Chromosomal Sex**	XY
**Inbreeding Estimate**	100.0% Inbred<br>(Percentage of the genome (autosomal and X chromosomes) that is homozygous or hemizygous for primary, secondary, and unknown backgrounds. See Genome Analysis)

Constructs Detected

BlastR	bpA	Cas9	chlor	cHS4	Cre	DTA	Flp	g_FP	hCMV_a	hCMV_b	hTK_pr	iCre	IRES	Luc	r_FP	rtTA	SV40	tTA
-	-	-	-	-	-	-	-	-	-	-	-	-	-	-	-	-	-	-

Refined Ideogram

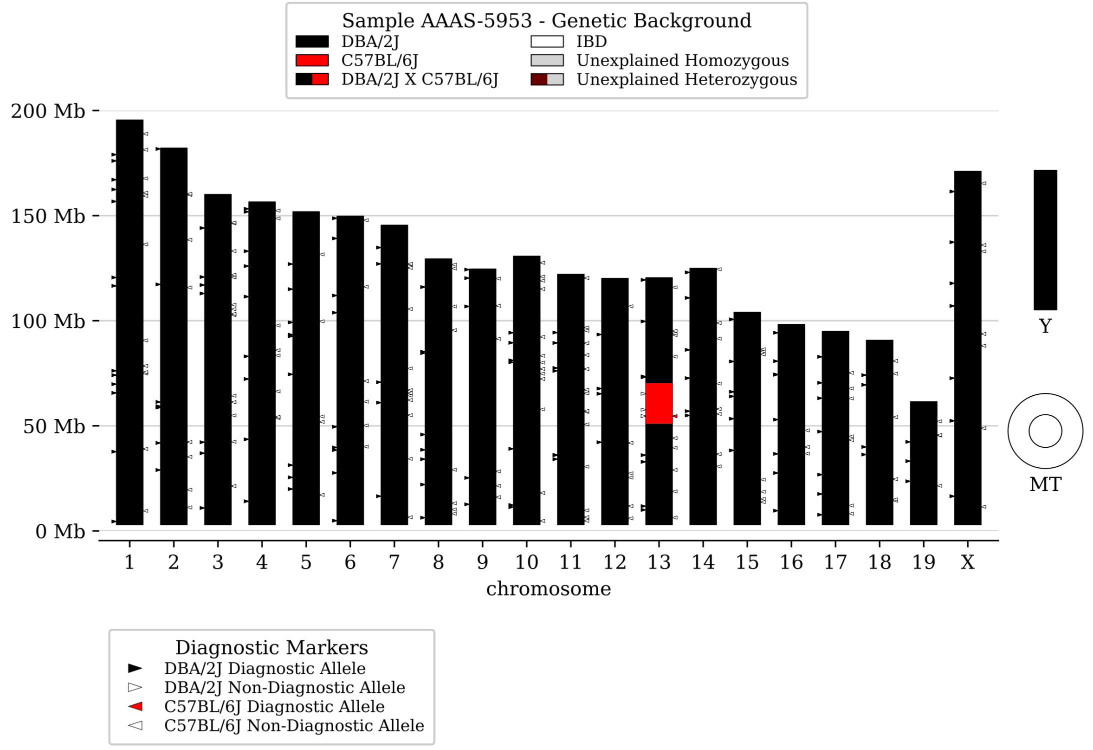

MiniMUGA Background Analysis v2.3.1

	Background	Zygosity	Informative Markers	Informative Markers %	Genome %
**Genome Analysis**	DBA/2J	Homozygous	2852	99.5%	99.2%
	C57BL/6J	Homozygous	15	0.5%	0.8%
		**Total**	2867	100.0%	100.0%

Y Chromosome

Y Haplogroup 9 - 100.0% Consistent
Includes DBA/2J and 21 other strains

MT Genome

MT Haplogroup 6 - 100.0% Consistent
Includes C57BL/6J, C57BL/6JEiJ, C57BL/6JJicTac, C57BL/6JRj, DBA/2J and 163 other strains

Backgrounds Detected (Diagnostic Alleles)

	Diagnostic Alleles Observed			
**Diagnostic Class**	**Homozygous**	**Heterozygous**	**Potential**	**% Observed**
DBA/2J	115	0	117	98.3%
DBA/2J, DBA/2JRj	22	0	23	95.7%
DBA/2J, DBA/2JOlaHsd, DBA/2JRj	3	0	3	100.0%
C57BL/6J, C57BL/6JEiJ, C57BL/6JJicTac, C57BL/6JRj	1	0	21	4.8%

Minimal Strain Sets Explaining All Diagnostic Classes (Number of Markers Explained):

- Solution 1: C57BL/6J and DBA/2J
  - DBA/2J: 140 / 143 (97.9%)
  - C57BL/6J: 1 / 21 (4.8%)
- Solution 2: C57BL/6JJicTac and DBA/2J
  - DBA/2J: 140 / 143 (97.9%)
  - C57BL/6JJicTac: 1 / 21 (4.8%)
- Solution 3: C57BL/6JEiJ and DBA/2J
  - DBA/2J: 140 / 143 (97.9%)
  - C57BL/6JEiJ: 1 / 21 (4.8%)
- Solution 4: C57BL/6JRj and DBA/2J
  - DBA/2J: 140 / 143 (97.9%)
  - C57BL/6JRj: 1 / 21 (4.8%)

MiniMUGA Background Analysis v2.3.1

	Chromosome	Start (Mb)	Stop (Mb)	Background	Zygosity
**Diplotype Intervals**	1	3000000	195471971	DBA/2J	Homozygous
	2	3000000	182113224	DBA/2J	Homozygous
	3	3000000	160039680	DBA/2J	Homozygous
	4	3000000	156508116	DBA/2J	Homozygous
	5	3000000	151834684	DBA/2J	Homozygous
	6	3000000	149736546	DBA/2J	Homozygous
	7	3000000	145441459	DBA/2J	Homozygous
	8	3000000	129401213	DBA/2J	Homozygous
	9	3000000	124595110	DBA/2J	Homozygous
	10	3000000	130694993	DBA/2J	Homozygous
	11	3000000	122082543	DBA/2J	Homozygous
	12	3000000	120129022	DBA/2J	Homozygous
	13	3000000	51136860	DBA/2J	Homozygous
	13	51136860	70449416	C57BL/6J	Homozygous
	13	70449416	120421639	DBA/2J	Homozygous
	14	3000000	124902244	DBA/2J	Homozygous
	15	3000000	104043685	DBA/2J	Homozygous
	16	3000000	98207768	DBA/2J	Homozygous
	17	3000000	94987271	DBA/2J	Homozygous
	18	3000000	90702639	DBA/2J	Homozygous
	19	3000000	61431566	DBA/2J	Homozygous
	X	3000000	171031299	DBA/2J	Hemizygous
	Y	0	0	DBA/2J	Hemizygous
	MT	0	0	IBD	Hemizygous
